# The Hospital. Nursing Section

**Published:** 1903-04-18

**Authors:** 


### The Hospital.

### Nursing Section.

Contributions for this Section of "THE HOSPITAL" should be addressed to the EDITOR, "THE HOSPITAL" NURSING SECTION, 28 & 29 Southampton Street, Strand, London, W.C.

No. 864.—VOL. XXXIV. *SATURDAY, APRIL* 18, 1903.

### Notes on News from the Nursing World.

### IMPORTANT CHANGES AT CROYDON INFIRMARY.

THE state of tension at Croydon Workhouse Infirmary has at last come to an end, thanks to the issue by the Local Government Board, of a new order, giving the Guardians powers not hitherto possessed by them. On Tuesday the full Board of Guardians endorsed the recommendations which had been formulated by a special committee. The most important of these are the appointment of a matron at a salary of £100 per annum, the person appointed to be a fully-trained and certificated nurse, competent to train probationers; the abolition of the office of the superintendent of nurses, and the consequent determination of the appointment of Mrs. Coburn-Coulton on the 24th of June; the abolition of the office of home sister and assistant to matron, involving the retirement of Miss M. A. Sheppard on the 24th of June; the appointment of a man and wife as steward and assistant matron, the latter to perform the domestic duties allocated to the matron by Article 47 of the new Order of the Local Government Board, dated March 23, 1903; and a resolution instructing the medical superintendent to draw up and submit to the infirmary visiting committee a scheme, showing the total number of nurses necessary to be employed throughout the infirmary, to enable the committee to assign the salary, or scale of salaries, to such nurses and submit it to the Guardians, and through them to the Local Government Board for approval.

### THE INDIAN NURSING SERVICE.

WE are informed that Miss M. Herbert, who has been appointed a member of the Advisory Board of the Indian Nursing Service, has not resigned her post as matron of Worcester General Infirmary, but retains it. We presume that her new duties will not take up much of her time, but in the case of Miss Loch, a similar arrangement has not, we conclude, been made. Miss Loch could hardly act at the same time as lady superintendent of an important hospital in India, and in an advisory capacity at home.

### PRINCESS CHRISTIAN AT IPSWICH.

As already announced, the formal opening by Princess Christian of the Ipswich Nurses' Home will take place on Saturday, May 9. Her Royal Highness will be accompanied by Princess Victoria of Schleswig-Holstein. She will arrive in Ipswich at 1.30, and will attend a luncheon at the Town Hall, which will be given by the Mayor to a number of invited guests. She will then proceed to the Nursing Home, where, after the opening, and unveiling a memorial to Queen Victoria, she will receive purses. Those from the county will benefit the funds of the Suffolk Nursing Association, and those from Ipswich that of the Nurses' Home. At a meeting of the subscribers to the Home the other day the rules recommended by the committee were adopted. A considerable discussion took place on an amendment that annual subscribers of half a guinea should have the privilege every year of giving two weeks' nursing and one week's nursing respectively to two poor patients who came under their notice, but the proposal was rejected.

### THEATRE NURSES AT A DUBLIN HOSPITAL.

THE need of the new operation theatre at the Adelaide Hospital, Dublin, is attested by the fact that though it has only been in use for a little more than six months, there have been a number of successful operations. Adequate provision does not, however, seem to have been made for the nursing staff. At the annual meeting of the hospital, it was stated that on the morning of that day there were seven operations in the theatre, and that a few nights previously the nurses were called upon at 10 P.M. to attend upon an operation which was not concluded till one o'clock the following day. In fact, Mr. Law who made these statements, said that the theatre nurses were overworked. This, however, can be prevented in the future by increasing the staff available for theatre work. Nurses are naturally glad to have the experience of attending operations, and therefore very slow to admit that their strength has been overtaxed. But in such an important institution as the Adelaide Hospital, there should be no risk of this kind.

### THE TRAINED WORKHOUSE MATRON.

AT a meeting of the Berkhamstead Guardians a very strong opinion was expressed against the appointment of a superintendent nurse in addition to the matron of the workhouse in their union. In this case we sympathise with the Guardians. The matron at Berkhamstead is a fully trained and certificated nurse, and therefore quite competent to act as superintendent nurse. The question of the result of the adoption of the Departmental Report upon the position of matrons who are trained nurses was also discussed at the annual meeting of the Association of Workhouse Masters and Matrons for Suffolk and Norfolk. In so far as the speakers objected to placing a matron who is herself a trained nurse in a position of inferiority to that of a superintendent nurse, we agree with them. But the training is the vital point, and it is curious that many who are indignant at the idea of the trained matron being in any way superseded by the superintendent nurse do not recognise the incongruity of an untrained matron being in authority over a trained superintendent.

### CHEMISTS AND NURSES.

A statement has been made in the *Pharmaceutical Journal*, by a correspondent who does not give his name, that nurses are in the habit of interfering with the chemist's customers and his charges, by recommending their patients to deal at particular "stores" on the ground of economy. Another correspondent follows it up by the assertion that the reason of the recommendation is not economy, but the fact that the "stores" in question probably allow a percentage to nurses on goods purchased. We dare say that nurses do often suggest that drugs may be purchased at a lower price than at the local chemist's, and where the means of the patients are limited, the suggestion is an act of kindness. But when patients are wealthy, there is no reason for the hint, and "live and let live" is a very good motto. As to the allegation that the suggestion is prompted by mercenary motives, we do not believe that this is at all generally the case. If it were so, patients and their friends would not have the implicit confidence they usually, with justice, repose in the integrity of the nurse.

### NURSING IN YORKSHIRE UNION INFIRMARIES.

A special nursing return has been issued for the East and West Ridings of Yorkshire by Mr. P. H. Bagenal, the Local Government Board Inspector. It shows that the number of sick nursed in 1902 was 4,812, that the nurses on day duty were 293, and the nurses on night duty 101, the number of patients to each nurse being 12·21. This is a distinct improvement, but Mr. Bagenal also has to state that the number of pauper attendants was 98, and that no fewer than 22 workhouses in his district are still without a night nurse.

### KINGSTON NURSING ASSOCIATION.

The date fixed for the opening by the Duchess of Albany of the new home of the Kingston Nursing Association in Birkenhead Avenue is June 8. Purses of the amount of £2 and upwards may be presented to the Duchess at the ceremony. Her Royal Highness opened the original home of the Association about ten years ago. This is used by the district nurses. The house lately purchased for the sum of £650, is for the private nurses, and is to be specially arranged for the benefit of patients who can afford to pay. The private nurses receive a salary of £35 a year with outdoor and indoor uniform, washing and board. In the second year they have, in addition, 1s. 6d. in every pound they are paid by patients, and in the third year 2s. A maternity nurse was appointed last year, and she appears to be doing excellent work amongst the poor. From May 1902 to January 1903 she paid 395 visits and attended 32 cases. The number of visits paid by the district nurses in the year was 15,868. The staff consists of three district nurses besides the lady superintendent, Miss Sawyer, and four private nurses. There is no doubt that the work of the Association is greatly appreciated, but, in this as in other instances, the policy of combining district and private branches in one organisation is open to criticism.

### NOTTINGHAM UNDER-NURSED.

Lady Maud Rolleston, who presided at the annual meeting of the Nottingham District Nursing Association, in impressing upon her hearers the need of more funds, signified her great sympathy with the movement. She went on to mention the work done by the twelve nurses and their superintendent during last year, showing that they had nursed 906 cases and that the poor had evinced their gratitude by giving donations amounting to £10 15s. But unfortunately the total of subscriptions received from the public, £800, was considerably short of what was required, and the expenditure of the Association had exceeded the income. In addition, the city of Nottingham was very much under-nursed, and at least three more nurses were required to deal with the great increase in the work undertaken. As matters were at present it was not unnatural that the nurses broke down occasionally, and quite recently the superintendent nurse, in addition to her own duties, had to undertake the charge of a district where the nurse's health had given way through overwork. Lady Maud expressed her opinion that in a large town like Nottingham, such a state of affairs ought not to exist.

### A DEFICIT AT BRISTOL.

The Bristol District Nurses' Society is still, after twenty-one years' existence, financially, in an unsatisfactory position. At the annual meeting, over which the Lord Mayor presided, it was stated that the deficit in the funds for 1902 was between £50 and £60. This is only a small sum to make up, and in view of the fact that the 18 nurses of the society attended 3,479 cases and paid upwards of 69,000 visits, its claims on the purses of the citizens of Bristol should not need to be pressed. But we make a suggestion to the society which may be deemed worthy of consideration. Formerly there were private nurses attached to the District Nurses' Home, but the committee, finding that the arrangement did not prove lucrative, relinquished it. They continue, however, to have "a daily nurse for paying patients." This is a distinction, but not much difference, and there doubtless remains an impression that the society is to some extent self-supporting. If the committee dropped the daily nurse for paying patients, they could appeal for support for the society on the ground that it was absolutely a charity, and received nothing whatever for the services rendered by the nurses. Meanwhile, we are glad to learn that on Saturday two performances of "The Liar" are to be given at the Spa, Clifton, on behalf of its funds.

### NORTHERN WORKHOUSE NURSING ASSOCIATION.

In the twelfth annual report of the Northern Workhouse Nursing Association mention is made of the heavy loss sustained by the Association during the year owing to the death of Mr. William Rathbone, who was one of its staunchest friends and most generous supporters. Unfortunately, in consequence of lack of funds—there is a deficit of £145—the number of the Association's probationers had again to be reduced last year. The staff on December 31st consisted of 83 nurses and superintendent nurses, and 11 probationers. Reference is made in the report to the improvement in the conditions of workhouse nursing, but an extract from the private letter of a nurse is cited as showing that much remains to be done. The nurse states that the infirmary is in a very bad condition and much needs repair. The doors, windows, and walls let in rain, and when the wind is at all rough the draught is fearful. These things, she says, have been reported, yet no one seems to care. But we can hardly believe that they have been brought before the Local Government Board inspector for the district, whoever he may be.

### LORD SHAFTESBURY AND DISTRICT NURSES.

It is pleasant to observe that Lord Shaftesbury is mindful of the traditions of his name, which will always be associated in the public mind with philanthropic endeavour. Moreover, his mother in her lifetime took a great interest in the work of the Society for Providing Nurses for the Sick Poor in the City of Belfast, on behalf of which he pleaded the other day. Lord Shaftesbury dwelt on the appreciation by the working man of the presence of a trained nurse in the home when his wife was stricken with illness, and he was absent all the day earning money to support the family. He testified to the self-denial and sympathy manifested by nurses generally and expressed his belief that the trouble they took, the time they gave, and the comfort they brought into the home could not be over-estimated. There is another reason why the Belfast organisation merits support. It is admirably managed, and has not only an excellent record as to the work done, but is in a financially sound position. This is a result upon which the ladies who are exclusively responsible for its management are entitled to be congratulated.

### SLOUGH NURSING FUND.

In the report of the Slough Nursing Fund which was read at the annual meeting, it is stated that with a view to economy and efficiency the committee made inquiries as to the advisability of affiliation with Princess Christian's Nursing Association at Windsor, but found that, in spite of the advantages which might result from the arrangement, it would be too expensive, and would also involve the adoption of an entirely different set of rules. The idea was, therefore, abandoned. Fortunately, the financial position of the fund has improved, and against a deficit of £5 18s. 6d. at the end of 1901 there was a balance of £20 18s. in hand at the end of 1902. Moreover, though there were some abnormal donations, the subscriptions increased to the extent of more than £5. The number of cases attended by the nurses was 112, and the number of visits paid exceeded 4,000. An effort is being made to raise an "endowment fund" of £2 000, which would ensure an adequate income, but the ordinary expenses should be mainly covered by subscriptions, and the next step should be the creation of a reserve fund.

### TRAINED NURSING IN CHINA.

According to a missionary nurse at a hospital in Nanking, there are few trained nurses in China. Many come, but only remain a year or two and then return home. Miss Hanzlik thinks that the cause is that a foreign nurse wishing to work among the Chinese must study the Chinese language before she is able to accomplish much among them, and, as she intimates, learning Chinese means prolonged study. When a trained nurse takes up executive work in a general hospital in China one of the problems to solve is where to get widows to train as nurses and Bible-women. This is because there are reasons why it is not advisable to employ married Chinese women in a general hospital. Another difficulty is that there is no demand for Chinese women nurses outside the hospitals, except for obstetrical nursing among foreigners in open ports. Miss Hanzlik states that the hospitals for women have girls in training both as nurses and physicians, with splendid opportunities for equipment, but her conclusion on the general question is that the Chinese people are not yet ready for trained Chinese nurses.

### THE IMPORTANCE OF SMALL SUBSCRIPTIONS.

In most parts of Cornwall, at all events, the work of Nursing Associations continues to be increasingly appreciated. At the annual meeting of the St. Austell Association, a highly satisfactory report was adopted, showing that 7,193 visits had been paid in 1902 by the nurses to 191 patients. The financial statement indicated the possession of a balance of £58 16s. 2d. at the bank. But the honorary treasurer, in referring to the balance-sheet, justly said that the Association depended too much upon large subscriptions, £113 2s. out of a total of £170 14s. being from 17 subscribers of £3 and above. The most stable foundation of all is a preponderating number of small subscriptions which, once secured, are not subject to the startling fluctuations of large contributions.

### A FIRST YEAR'S RECORD.

The first annual meeting of the Ormskirk and District Nursing Association has been held, and the report submitted to the subscribers showed that the income exceeded the expenditure to the extent of £75 13s. 4d. The nurse, who has only been at work six months, attended 1,255 cases, and it was stated by several speakers that her work was already greatly appreciated by the poor in the town. Dr. Heald stated that the Ladies' Charity, which had previously existed, had been greatly abused, but that the Nursing Association, which had taken its place, was more than a substitute for it.

### A NURSE IN AN IRISH RAILWAY ACCIDENT.

There was a serious railway accident, in the small hours of Saturday morning, to the night mail on the Midland Great Western Railway between Ballymoe Station and Castlerea. Among the passengers was a nurse belonging to Steevens' Hospital, Dublin, Miss Taafe, who, being happily unhurt herself, was able to render valuable aid to the wounded, both before and after the arrival of the doctors. The readiness with which she volunteered her services, and their efficiency, was warmly acknowledged.

### SHORT ITEMS.

The s.s. *Ionian*, which arrived at Southampton from South Africa last week, had on board Nursing Sisters M. Mackintosh, S. Brown, and A. Blair of the Army Nursing Service Reserve.

### The Nursing Outlook.

"From magnanimity, all fear above;
From nobler recompense, above applause,
Which owes to man's short outlook all its charm."

### NURSES AND SETTLEMENTS.

In America the connection between the nurse and the Settlement is much closer than in England: indeed, many nurses may never have come into contact with Settlement work here. A Settlement is an institution intended to gulf the chasm between rich and poor—between East and West. The first Settlement started in London was Toynbee Hall, Whitechapel, and the idea was that the young University man was to live there, and to try and pass on to the working man some of the culture gained at college. The success of Toynbee Hall was great, and the women promptly started a Settlement in Southwark on similar lines, while Mrs. Barnett at Whitechapel added women's work to Toynbee Hall. And then Settlements developed widely; they sprang up at Liverpool, Glasgow, and elsewhere, and the discontented young lady learned to wear off her *ennui* by going and dwelling amongst the poor and telling them how uncultured and unthrifty they were; and then after a few months, returning in virtuous exultation to the luxuries of the West End, and imagining a halo about her head.

But in America the Settlements are run more on business lines, and probably the most noticeable and the most original, is the Nurses' Settlement in Henry Street, New York. It was founded by Miss Lilian D. Wald, who, after graduating as a nurse, went to live on the East side with a friend, and give her services to the poor. They found the demand for their aid greater than they could supply and they persuaded other nurses to join them, and then a gentleman came forward and supplied these devoted women with a house in which to dwell. And with the house the work expanded, a little garden at the back became a playground for the children, a room in the basement became a dispensary, and a big dining-room was used at nights for the meetings of boys' clubs, for lectures and so on. The Settlement began by what we know as "district nursing," and then grew into the more varied labours common to English Settlements. There is but little room to tell here how a country house for the ailing and the children, how a school house and other branches were added on; but President Roosevelt and Mr. Jacob Reis know all about the Nurses' Settlement and have learnt many lessons from it. There they heard of the need of playgrounds for the children, there they heard of the defective eyesight in the schools: of all the numerous citizens of New York who have done good work, we defy any to show a record to beat that of Miss Lilian D. Wald. She lived amongst the people and got to know their needs, she had the gentle sympathy of a cultured lady, and the power of repression of a trained nurse; she was a connecting link between the voiceless poor and the powerful senator, and now her works arise and call her blessed. And the lesson we would learn from this is that the nurse in England might be much more useful as a citizen than she is at present, especially if she were brought more into contact with Settlement work. The first idea of a Settlement here, according to Canon Barnett, is "a group of persons who recognise the obligations of citizenship." And so you get lecturers on Economics as at the Passmore Edwards Institute, or Sister Kerrison of the Canning Town Settlement sitting as a guardian, and Sir John Gorst and Sir Charles Elliot learning their practical statesmanship at Toynbee Hall. But at the present time there is a decided fall in the number of Settlement workers both in London and the provinces, especially amongst women. Slumming is going out of fashion, and doubts as to the result of salvation by personal pettifogging are beginning to arise. The slums seem no better for the visits of ladies in their silks, and the costers seem no more cultured because of Dr. Kimmins' University Extension lectures at Bermondsey. And yet we have not the hardihood to let these things be; we dare not pass by on the other side so long as London remains the most miserably poverty-stricken, the ugliest and the cruellest, and yet the most wealthy city in the world. It seems to us that the touch of human sympathy, the love that never faileth, might perhaps be introduced more into Settlements if they dealt more with the care of the sick on practical lines—if they had more nursing members, and indeed made nursing the poor and the care of the little ills of little children their main concern. A hardness of heart is inevitably engendered in the rich person who goes to dwell amongst the poor with the direct intention of telling them of their faults. There was a book published some years ago by the Scientific Press called "Charity Organisation and Jesus Christ," which was one of the most powerful indictments of the professional faultfinder we have ever read; it proved that the person who dealt with his fellows without love was bound to fail, was necessarily anti-Christian. And this is the lesson the Settlements are now learning in England. They might learn the way out of their troubles by a visit to the Nurses' Settlement at New York, where the help is ready, the service is practical, the sympathy is great. There is no condescension, no airing of superiority, no mere scolding of the poor for their poverty. Instead there is the daily service, the constant realisation of the evils and meeting them, not by grumbling at those who are the slaves of their environment, but by passing the knowledge gained on to the law-makers, so that the environment may be amended. Only practical and sympathetic persons can do this, and therefore we believe that if the London Settlements, instead of their interminable lectures and priggish visitors in silks and feathers, would number more nurses amongst their members, they would pass through the present season of depression and come to the fulfilment of their aims.

### Lectures on Ophthalmic Nursing.

By A. S. Cobbledick, M.D., B.S.Lond., Senior Clinical Assistant Royal Eye Hospital, late House-Surgeon and Registrar, Royal Eye Hospital.

LECTURE VIII. — GENERAL HINTS ON NURSING (*cont.*).—DRUGS AND DRESSINGS AND THEIR APPLICATION.

Another source of danger to an operation case, both directly and indirectly, is a dust-laden atmosphere. The prevention is to be found in removing dust without allowing it to be disseminated, and this can only be satisfactorily carried out by means of *moisture*; no attempt should be made to remove dust without a well *damped* duster. This may appear to be a small detail, but it is an important one.

There is ample scope for attention to detail in ophthalmic nursing, from the way a pad and bandage are applied to that necessary for the successful issue of an operation for acute glaucoma. One of the most important duties a nurse has is the instillation of drops into eyes; in this connection she should never use a drop bottle which is not labelled, or, if labelled, never instil drops without reading the label: never pass a drop bottle to anyone else for use without acquainting yourself of the contents and their strength. Inattention to this detail has been the means of spoiling a good eye, through instilling some strong caustic instead of cocaine.

The nurse's patience will be frequently on trial, *e.g.* in nursing a senile patient who has been operated upon for cataract or glaucoma; constant watchfulness is necessary to prevent the removal of dressings or digital manipulation of the affected eye by the patient.

These are but a few examples of the many traits in a nurse's character which will be tested in ophthalmic work.

Drugs Used Locally in Ophthalmic Practice.

The most common are cocaine hydrochloride in solution (distilled water) 4 per cent. and 8 per cent. Atropine sulphate gr. ii. or gr. iv. to the ounce of distilled water.

Homatropine hydrobromide, gr. iv. ad. ℥i.
Eserine sulphate gr. $\frac{1}{3}$ to grs. ii. ad. ℥i.
Adrenalin 1 in 10,000 parts of water, or stronger.
Salts of silver, eq. nitrate of silver.
Euphthalmine, gr. iv. ad. ℥i.
Fluorescine.

*Cocaine* is a most useful drug in ophthalmic surgery; in 8 per cent. solution it produces total loss of sensibility to such a degree that major operations on the eye can be performed after three applications, rendering the use of a general anæsthetic unnecessary. It is also useful in removing foreign bodies from the cornea or conjunctival sac, in certain painful affections of the eye, and in its dilating action on the pupil; in such cases a 4 per cent. solution is sufficiently strong.

Sulphate of atropine is chiefly used in inflammatory conditions of the iris and ciliary body; it dilates the pupil, renders accommodation for near objects impossible by paralysing the ciliary muscle, and so gives *absolute* rest to the inflamed parts of the eye. It is also largely used in another connection, viz., in children and young adults before estimating an error of refraction, *i.e.*, as a preliminary to ordering glasses.

Homatropine and euphthalmine have a similar action to atropine, but their effect is less lasting.

Eserine has the opposite effect of atropine, as it renders the pupil very small; its use is chiefly confined to the treatment of certain corneal troubles and a disease called glaucoma. Adrenalin is a comparatively new drug which has found considerable favour in ophthalmic practice on account of its usefulness in stopping bleeding, *e.g.* in operations on the eyelids where the bleeding may be so free as to interfere with the operation; it has also been used in certain inflammatory troubles of the eye, but in this connection it is still on its trial.

Fluorescine is used on account of its staining properties; ulcers of the cornea difficult to define are readily stained, and their shape and size perfectly outlined.

Other less common drugs will be mentioned in treating of diseases of the eye.

Many different forms of drop-bottles have been devised for the instillation of drops into the conjunctival sac; perhaps the best is that which allows the drops to escape slowly through a piece of capillary glass tubing on inverting the bottle, and which is fitted with a glass cap to prevent dust from settling about the mouth of the orifice. When applying drops it is important for two reasons not to allow the glass tubing to come in contact with any part of the eye, firstly, because the cornea and conjunctiva are extremely sensitive; secondly, to avoid the possibility of carrying any infection from one eye to another. If by any mischance the glass tubing comes in contact with a purulent discharge, it should be removed, cleaned, and boiled.

Of course, oily preparations cannot be administered in the above way; it is customary to use a bottle fitted with a tapering solid glass rod. The obvious disadvantage of this method is that the lack of fluidity of the oil may tempt one to touch the eye with the rod in order to hasten the instillation, and in this manner infection might be carried from one eye to another.

In the case of young children drugs are applied to the eye through the medium of ointment, as the process is easier and the result more satisfactory; it is best applied with a camel hair brush or the end of a taper or used wax match; the lower lid must be drawn down with the thumb and the ointment placed on the conjunctiva of the lid.

*Dressings and their Application.*—This is an important branch of ophthalmic nursing, and a thorough acquaintance with the subject can only be obtained by practical experience.

Although all dressings applied to the eye should be *aseptic*, it is not usual to use antiseptics to the extent that they are used in other branches of surgical practice; anything approaching a *strong* antiseptic applied locally may make its way through the closed lids, irritate the conjunctiva, make the patient uncomfortable and restless, and possibly spoil the result of an operation.

An ideal dressing should be aseptic, comfortable to the patient, and applied so as to exert a firm and uniform pressure on the eye; to a certain extent the lids act as a splint, but they have to be kept closed, and this is one of the chief uses of a pad and bandage.

The comfort of the patient must be sought by keeping eyelashes and other foreign bodies out of the conjunctival sac, and by applying a greasy or wet dressing to the closed lids. Generally speaking, dry dressings are irritating and uncomfortable to the eye. If a wet dressing is used, there is nothing better than a thin pad of absorbent wool damped in a weak solution of boracic acid (gr. x. ad. ℥i.). It is sometimes advisable to use a dressing of boracic lint, kept cool and moist by frequent immersion in iced water: in such a case a bandage is not applied.

When a greasy dressing is applied next the lids, a circular stiff piece of muslin, large enough to project about a quarter of an inch beyond the orbital margin, is smeared with carbolised vaseline and carefully applied. Over this a pad of absorbent wool, thinner in the centre than at the margin, is fixed with a turn of bandage; the bandage should be about two inches wide and either of flannel or bleached calico.

### Words of Advice to Nurses.

BY ESTHER H. YOUNG.

### LECTURE IV.

*Concluded from page* 19.

When you get your promotion, remember you are put over "pros.," and you are expected to teach them and they must be obedient to you, and treat you with that respect to which allusion has been made. But you, too, have your part; be patient with your subordinates, even as you expect your superiors to be patient with you. Avoid flattening remarks such as these—"I've told you so before," "I have shown you how to do this heaps of times." Teach them how to wash a patient, how to clean other people's nails, how to "do heads," how to make beds and shake pillows without shaking the patient. Show them how to measure medicine accurately, how to take temperature, pulse, and respiration. But though with firmness and decision yet let all this teaching be done with patience and gentleness; remember that they really do not know, and even forgive them if they have forgotten. Instil into your young "pros." such a spirit of care that they will really give their mind and attention to the details of their work, and so become less careless or forgetful. So much depends on the sister of the ward; the responsibility of her example is very great, and her influence affects the whole tone of the ward. Each has a little world of her own. So many sick-folk with bodies, souls and minds to be cared for; doctors, senior and junior, to work for and with; patients' friends and relations; other visitors; head nurses and "pros." They are responsible for the well-being of all, and must take the blame of all mishaps, big and little, which occur in their domain. This their juniors do not always realise; but it is so, just as the matron is responsible for the well-being of the whole, and is to blame for all errors and accidents in nursing throughout the hospital.

Think of this now, and remember it later when you are a sister. Have patience and tact then in managing your nurses. If a sister is careless, all the nurses in the ward will be the same. If a sister is too familiar with the men, all the nurses will follow. If "Sister" does it, it ought to be right. If Sister wastes things, the nurses of course will do the same.

Now to those of you who, when trained, become private nurses. Go forth to nurse people in their own homes with the firm intention of entering into the lives and interests of each man, woman or child to whom you are sent, really caring for him or her. "I wish I was not going to be a private nurse." "I shall hate private nursing, and I am not going to read to those sort of people, and curl their hair and fad them up."

"Those sort of people," though not hospital patients, need as much and even more care of their bodies, souls and minds. When you go into a house be ready to do anything for your patient, anything that will help him to recover or add to his comfort. Don't be a smart right hand to the surgeon and on your dignity at all other times. Make friends with the cook, speak politely to the maids, consult the near relations of your patient as to likes and dislikes. Win the whole house. This is part of private nursing, and in so doing you will much more easily win and so manage your patient. Be ready to read anything to him or her; be ready to curl her hair, or tie his ties; to go out with them, to shop for them. Take your hospital knowledge, but drop the "red tape" necessary to an institution. Don't look upon relations as "pros." and servants as ward-maids. Nurses may do so much good in a house, but many do so much harm. Be tactful, be kind, be humble, be patient, be strictly obedient to the doctor under whom you work, and never discuss his treatment. Never talk of one patient to another, nor of anything the patient tells you to others in the house. All they say to you must be held by you as sacred. Interest them, if well enough, in all that goes on, and take an interest in their work or special talent, or whatever they care about most. Don't talk in your patient's hearing of him or her, don't show them that you think their cases "slow," never tell them they are hysterical though you may think so. Or, if you take up district nursing, remember an Englishman's cottage in his castle.

You must insist on cleanliness. You may have to encounter much dirt and perhaps rudeness, but the latter is not often the case; go gingerly to work; don't listen to the unpleasant remarks; don't show disgust about the dirt, but get rid of it all the same. Sweep, dust, scrub, and let them think it is just a fad of yours. Be interested in the children and in the work. Be firm on certain necessary points for the patient, but don't ride rough-shod over the bread winner or "missus." Humour them in little things. Their gratitude is so touching—if you seem to do very little good sometimes because they in their ignorance will undo all your work, yet that little, and the presence of nurse may do so much to brighten, help to refine their lives. Don't get tired of those endless legs which never get well, or grannies with chronic bronchitis, or paralysed crochetty "daddies," don't compare them with your cases in hospital. Enter into the lives of each one, and try to care for them sincerely. In conclusion—to one and all—private or hospital nurses—junior "pros." or sisters—let me earnestly recommend carefulness in speech. My dear friends, I beseech you, I entreat you, be careful what you say. Never by any chance repeat what you hear about anyone. We all say foolish things and make unguarded remarks and dissect each other's characters.

In our haste how often we say unkind things! Once said, they may do very little harm—repeated even to one person—one supposed safe person—may do untold harm. These repetitions are hardly ever quite accurately given, and it is most dishonourable to listen to tales. It is intentional mischief making when we carry tales from one to another. "Listening to the latest" is such a great temptation in Hospital life, and "listening to the latest," or repeating the "latest" may work the ruin of a life. The temptation yielded to in early Hospital days grows. As seniors the habit is incurable, and the harm done by it is incalculable. Avoid chattering. If you think it necessary to report to a senior about a fellow worker, be honest, and first tell that fellow worker what you intend to report. You may find that what you intended to report to that superior was utterly untrue. Give your fellow worker a chance to speak for herself. Be straightforward, and you will be saved from doing untold harm.

By these few words I have tried to help those who aim at becoming nurses, in the highest sense of the word, and I would conclude by reminding you of the key-note of a true nurse's life.

It must be charity—love. I would impress this the more strongly because I think the temptations against perfect charity are even greater for those in training than for others outside Hospital. Nothing so mars hospital life as this lack of charity in pupils or teachers.

"Therefore give us Love."

### Everybody's Opinion.

[Correspondence on all subjects is invited, but we cannot in any way be responsible for the opinions expressed by our correspondents. No communication can be entertained if the name and address of the correspondent are not given as a guarantee of good faith, but not necessarily for publication. All correspondents should write on one side of the paper only.]

### HOW TO MAKE UP A FIRE.

"The Sister-in-Charge of the Children's Hospital, Gloucester," writes: In The Hospital of April 4th there is a description of how to keep up a fire quietly. Perhaps nurses would like to know that for 1s. 6d. we supply by post a wooden shovel and poker for that purpose.

### NURSING IN BIRMINGHAM.

"A Private Nurse" writes: There is a great want felt of a nurses' hostel or home for private nurses between their cases in Birmingham. Although there is a large nurses' co-operation, as well as a great many smaller ones, no home is provided for the nurses. Surely a hostel on the same lines as the one in Frances Street, London, would pay well. Many nurses here would realise the comfort of such an institution and will agree with me in thinking how very lonely and cheerless all apartments are after a trying and anxious case. I sincerely hope that someone will be enterprising and start a hostel in Birmingham.

### THE NEW SECRETARY OF THE ROYAL BRITISH NURSES' ASSOCIATION.

"Miss G. A. Leigh" writes with reference to our note on the appointment of Miss Annie Hobbs as her successor in the secretaryship of the Royal British Nurses' Association, to point out that the private nursing element in the Association is an important one and entitled to consideration, but it is only one strand among many. "The Association," she adds, "numbers among its members matrons, sisters, and nurses from among the principal hospitals in the Empire, as well as the leading Poor Law Infirmaries, a large number in addition are in charge of Isolation and Cottage Hospitals and Convalescent Homes. Others are in Queen Alexandra's Imperial Military Nursing Service, hold appointments under Colonial Governments, or are working in the mission field. Yet others are engaged as district nurses, and many are occupied in philanthropic institutions such as orphanages and Homes of Mercy."

### SUPERINTENDENT NURSES AND THE MIDWIFERY CERTIFICATE.

"Superintendent Nurse" writes: I am glad that the Matron of Warwick Workhouse has given utterance to the thoughts of others who have read the report of the Departmental Committee regarding the superintendent nurse holding a midwifery certificate. I also received my training in a general hospital, and did not hold a maternity certificate when I took my present post, but after being here some months took my L.O.S. certificate. It is impossible to take a maternity certificate without severing one's connection with the general hospital in which one is working. My opinion is that it is much more important to have a superintendent nurse who has been trained in a general hospital than one who holds a maternity certificate, if one with both cannot be had, as I know the value of the former in training probationers. The nursing in the sick wards of a workhouse is principally of chronics, which must limit the scope of a nurse's work; therefore, if the superintendent nurse is trained in a general hospital, she can give the nurses, from her own practical experience, a much fuller training than one who has nursed few acute cases, and so equip them better for facing whatever nursing they may take up when leaving their infirmary. To any one of average ability it is easy to qualify herself for the L.O.S. examination, and she need have no difficulty in getting her cases as they are on the premises. I hope the Local Government Board will alter this clause in their report of the Departmental Committee before making it compulsory, as otherwise they will not attract nurses with a general training to fill their posts.

### MIDWIFERY TRAINING.

"One who Trained at the Hospital" writes: One sees so often nurses asking for information where they can get free or reasonable training in midwifery. Everyone should know there is not any such thing as a free training in this work, but some of your readers may be glad to know that by paying for their board they can get their training free in return for giving their services at the "Hospital for Soldiers' Wives," Woolwich. The course is for six months. The training is excellent, both in practice and theory. I feel sure that there are many nurses who would like to know of this, and who cannot afford the high fees connected with the work, and yet who wish for, and must have, this training. It has so often occurred to me what a boon it would be to nurses if the workhouses and infirmaries would throw open their lying-in wards on similar terms as those already mentioned. I fear that nurses are not a saving class, and it is often a very hard matter to get the money together for this training, and I feel sure that they would greatly appreciate any point stretched to enable them to get this training on reasonable terms.

### POST-GRADUATE TEACHING.

"Miss Evelyn L. C. Eden," Kingston Grange, Taunton, writes: Will you allow me to make a few remarks on the subject of the interesting article which appeared in a recent issue of your paper on "Post-graduate Teaching" for nurses? I have long felt the necessity for this most strongly. But it appears to me that it is in practical work even more than in theory that the trained nurse requires "freshening up." The theory she can, to a certain extent, acquire from books and such papers as The Hospital, though, no doubt, lectures (implying a lecturer who will answer questions) are more instructive. But the routine of asepsis, operative or general surgical work as well as many methods of treatment, she can only acquire in the wards of a hospital. Until the hospitals will take the matter up and allow nurses to go back to the wards for a month or so at a time it will be almost impossible for district and private nurses to keep pace with the newer methods. I venture to think that this arrangement need not necessarily disturb the routine of hospital work to any unreasonable extent. The nurses in question could either take holiday duty free (and be taught by the sisters or simply go round the wards like a medical student whilst the work is being done. This should be sufficient for a thoroughly trained nurse, and she would doubtless be willing to pay a reasonable fee for the privilege. In either case it would be an economy to the hospital, and the staff would have to act loyally to the post-graduate pupil and endeavour to make it easy for her to acquire information.

### Wants and Workers.

Will any kind friend having a warm disused dressing-grown or cloak allow District Nurse, Haveringland, near Neepham, Norwich, to have it, for an aged woman, very poor, crippled through rheumatism, and unable to be dressed?

Will any lady or gentlemen having any disused appliances for nursing or undergarments kindly help a district nurse on a large and scattered district? Old linen thankfully received. Address parcels to Nurses' Home, Chapel Street, or to Mrs. Robinson, Hon. Sec., School House, Skinningrove, R.S.O., Yorks.

### Appointments.

[No charge is made for announcements under this head, and we are always glad to receive, and publish, appointments. But it is essential that in all cases the school of training should be given.]

Bolingbroke Hospital, Wandsworth Common, S.W.—Miss Jeannie Rennie has been appointed matron. She was trained at the London Hospital, and has since held the appointments of staff-nurse and sister in the same institution.

City Fever Hospital, Liverpool.—Miss Gertrude Eva Heald has been appointed charge nurse. She was trained at the Bradford Union Hospital, and has since been on the private nursing staff of the Queen Victoria Surgical Home, Wolverhampton, staff nurse at St. Mary's Infirmary, Upper Holloway, N., and ward sister at Woolwich Infirmary.

City of London Hospital for Diseases of the Chest, Victoria Park, E.—Miss Stuart Cameron has been appointed assistant matron, and Miss Isabel Rippon night sister. Miss Cameron was trained at the Dumfries and Galloway Royal Infirmary, and has since been sister at the Children's Hospital, Cardiff, day and night sister at the Royal Infirmary, Preston, and home sister at the Union Infirmary, Fir Vale, Sheffield. Miss Rippon was trained at the Royal Infirmary, Wigan, and has since been sister of the male wards and theatre at the County Hospital, Newport, Monmouthshire, and sister of the phthisical wards at Southwark Infirmary, East Dulwich.

Hospital and Home for Incurable Children, Maida Vale, London.—Miss Isabel E. Forster has been appointed matron. She was trained at Dover General Hospital, and the Royal Orthopædic and Spinal Hospital, Birmingham, and has also been house-matron and nurse at the West Riding Home for Women, Wakefield, charge nurse at the Royal National Hospital, Ventnor, matron at St. Mary's Cottage Hospital, Tenbury, and matron at Rosehill Hospital for Sick and Incurable Children, Torquay.

Incorporated Belfast Maternity Hospital.—Miss Kelly has been appointed matron. She was trained at Birmingham Infirmary, and has since been sister in the same institution.

Leeds Union Infirmary.—Miss Mary D'Evelyn has been appointed matron. She was trained at the Children's Hospital, Belfast, where she was afterwards charge nurse, and the Birmingham Infirmary, where she was subsequently sister. She holds the L.O.S. certificate.

Northern Hospital, Winchmore Hill, N.—Miss Emily Mearns has been appointed housekeeper, and Miss Isabel Elders night superintendent of nurses. Miss Mearns was trained at the Royal Hospital for Sick Children, Aberdeen, where she remained on the hospital and private nursing staff for seven years. Miss Elders was trained at Guy's Hospital, London. She has since been charge nurse at the Northern Hospital, Winchmore Hill, for several years.

Richmond Union, Surrey.—Miss Emily Rogers has been appointed night nurse. She was trained at Wandsworth and Clapham Union Infirmary and has held posts at Halstead Union Infirmary and Epsom Union Infirmary.

Royal Hospital for Sick Children, Glasgow.—Miss Julia Simpson has been appointed matron. She was trained at the Royal Hospital for Sick Children, Glasgow, and King's College Hospital, London. She has been sister at the Children's Hospital, Liverpool, and at the Royal Infirmary, Bristol.

Staffordshire General Infirmary.—Miss Mary L. Parker has been appointed night sister. She was trained at the Swansea General and Eye Hospital, and was afterwards sister at the Royal Victoria Home, Bristol, and sister at the Royal Cornwall Infirmary, Truro.

### Presentations.

East Sussex Hospital.—In view of the approaching marriage of Miss Barbar, matron of the East Sussex Hospital, Hastings, she has been presented with a set of silver entrée dishes from the nursing staff. The following is the inscription: "To our dear matron; a small token of love and respect, with every best wish for future happiness." Other tokens of the esteem in which Miss Barbar is held, are presents which she has received from the house surgeon, the chaplain, and the maids and porters.

Steyning Union Infirmary.—Miss A. M. Brown, who is leaving Steyning Union Infirmary in order to take up similar duties at Stockport Infirmary, has been presented by the probationers with a cowhide leather bag in appreciation of the kindness and consideration she has shown towards them the past year.

### "The Hospital" Convalescent Fund.

The Hon. Sec. acknowledges with thanks the receipt of 2s. 6d. from Miss Elise Jonil.

### TRAVEL NOTES AND QUERIES.

From Neuchâtel to Interlaken (Sister Maud).—I find the quickest way for you from Grandson is viâ Yverdon, Neuchâtel, and Berne. Circular ticket from London viâ Calais, Paris, Neuchâtel to Yverdon, thence to Berne, Interlaken, and Meiringen, returning viâ Berne, Paris, Calais, and London. Cost, through Messrs. H. Gaze and Sons, 53 Queen Victoria Street, £7 12s. 9d. This would enable you to touch at Grandson as well as Yverdon.

St. Servan and Bay of St. Malo (M.E.R.).—You are quite right, it is charming, but it has often been recommended in these columns, and articles were written on it in '99. The Maison Mathias has often been mentioned by me and is all you say. Thanks for all particulars, personal recommendations are always valuable.

Passage to Canada (E. A. W.).—Your query arrived too late for attention last week. I send you a list of the different lines with their prices, and should be obliged to you to return it to me for I have only a limited number. You had better write to Messrs. Cook when you have chosen your route *in good time*, because very often all the berths are booked already in the steamer you choose. I also send you a yellow paper of instructions which you may keep and which will be useful. Be careful to attend to the measurement of your *cabin* luggage. Let me recommend you to take a good wrap for sitting on deck, it is often intensely cold even in May. Cloaks are not so suitable as warm, long coats. Take also a soft hood or Tam o'Shanter, a uniform bonnet and veil would be in the way in a high wind.

The Two Candebecs (Doubtful).—I do not think you at all a nuisance. If all were experienced travellers there would be no employment for me. It is Candebec near to Yvetot of which I spoke to you. When terms are quoted *en pension* it includes everything; bed, room, and food. If you are thinking of many excursions ask the landlord to allow you to take something with you for lunch instead of being in to the midday meal. They are generally obliging in this matter. Do not forget the name I recommended to you, Hôtel de la Marene.

### The Nurses' Bookshelf.

PRAYERS FOR HOSPITAL WARDS. By A. B. (Kidderminster: Hepworth. 2s. post free.)

THIS volume of prayers is compiled by a matron who has probably felt the need of one for use in hospital wards. They seem admirably adapted for the purpose, and contain short and appropriate prayers for the morning and evening of every day in the week, and a brief portion of scripture.

LETTERS TO MOTHERS. By F. M. H. L. (Cable Company, Great Queen Street, W.C. 1s.)

THESE three letters of advice to mothers on the important subject of their physical and mental condition as affecting the future well-being of their unborn children are written by one who has deeply at heart the welfare of her sex. While expectant mothers may not be able to follow all the rules laid down for their direction, we feel sure that much benefit will be derived from a perusal of the suggestions and words of counsel offered to them during their months of waiting.

OPERATION NURSING INSTRUCTIONS. By F. W. COLLINSON' M.D., F.R.C.S.Edin., M.R.C.P.Lond. Second Edition. (Bristol: John Wright and Co.; London: Simpkin, Marshall, Hamilton, Kent, and Co., Limited. Copyright. Price 1s. per dozen, 7s. 6d. per 100, post free.)

THESE are a set of good general rules for the nursing management of operations performed in private houses, and when adapted or corrected to the requirements of individual cases and the individual idiosyncrasies of medical practitioners should prove valuable aids to both doctor and nurse. As the directions are compressed into the space of what would correspond to three sides of a large sheet of note-paper they can be easily carried in a purse or pocket-book. It is made clear that these rules are for the use of medical men who may like to give them to the nurse in charge of the case he is about to operate on, and not for the nurse to follow before taking his instructions.

THE HOSPITAL SERVICE BOOK, CONTAINING SHORT SERVICES AND HYMNS FOR USE IN THE WARDS OF HOSPITALS AND INFIRMARIES. Revised and Enlarged. By CHARLES PARKHURST BAXTER, M.A., Chaplain to the Hospital for Women, Soho Square, etc. Dedicated by permission to the Lord Bishop of London. (London: Henry Frowde, Amen Corner, E.C. Price 2s.)

THIS little book will be of great help, not only to chaplains, but to sisters and nurses who feel their responsibility towards the souls as well as the bodies of their patients. Besides simple services for morning and evening prayer, there are prayers and hymns suitable for every contingency of hospital life. The prayers are for the most part taken from the Prayer Book, with a few from ancient sources, so that the services will have the advantage of familiarity to many of the patients, while the omission of some features and the inclusion of others will prevent their being too fatiguing, and will render them more fitted to the special circumstances of those who join in them. If there is anything to which we might take exception, it is to the children's services, which, though beautiful in themselves seem to us a little too elaborate for sick children, some of whom have had little if any religious training before they were brought to the hospital. For the benefit of the children also we should welcome a larger selection from the "Sacred Songs and Solos" and other collections of bright hymns, even though their tunes may be catchy. Catchiness is not to be despised when dealing with children, or even with adults of the class from whom hospital patients are largely drawn. The book will, without doubt, carry help and comfort to many a sick-bed, and is as well suited for private use as for the wards of a hospital.

### For Reading to the Sick.

"NOT MY WILL, BUT THINE BE DONE."

To the still wrestlings of the lonely heart
He doth impart
The virtue of His midnight agony,
When none was nigh,
Save God and one good angel, to assuage
The tempest's rage.

"O Father! not My will, but Thine be done!"
So spake the Son.
Be this our charm, mellowing Earth's ruder noise
Of griefs and joys;
That we may cling for ever to Thy breast
In perfect rest!

*Keble.*

Perhaps of all the trials which a loving and merciful Father permits His children to undergo, there is none greater than that sinking of heart and lowness of spirit which they are called at times to suffer. Many think their unhappy state is a sign that God is angry with them, that they have sinned past forgiveness, that they have no faith and no love, or even that they cannot be saved. All this is most false. It is measuring God by our own poor weak feelings. It is like saying the sun has ceased to shine, when there is a little cloud over our heads. We know very well that, if we could only look through the cloud, we should see the sun shining in all his brightness and glory beyond. And so too we ought to believe that, if we could look through our own darkness and dimness of spirit, we should see God's love shining over us in all its warmth and splendour still. O do not let us believe our own feelings rather than God's word! Let us try to say, "I cannot feel happy; I cannot help being downcast and afraid; but yet I will believe through all that God loves me." "Though I am sometime afraid, yet put I my trust in Thee." O loving Father, I am Thine. Do "with me what Thou wilt." Then wait, and be patient. Remember the happy promise, "Heaviness may endure for a night, but joy cometh in the morning." And think this, "The night is far spent: the day is at hand."

*Anon.*

O fear not, though before thee lies
A dark and narrow way,
For at thy side thy Saviour walks,
Thy Comforter and Stay.

Hold fast His hand, and lean in faith
Upon that mighty arm;
His love and power will guide thy steps,
And shelter thee from harm.

The Resurrection and the Life
Be Thou to us, O Lord,
Fulfil to us the gracious pledge
Of Thine own blessèd Word.

*C. Wordsworth.*

### Echoes from the Outside World.

### Movements of Royalty.

BEFORE King Edward left Lisbon last week he handed over £4,500 for the poor of the city as well as many gifts for officials. Upon arriving at Gibraltar the church bells rang a welcome, the ships in the harbour were dressed with flags, and salutes were fired. The weather fortunately was superb, and all the way to the Convent—the residence of the Governor—troops and large crowds lined the streets. The King stopped en route and shook hands with two Crimean veterans of the Fusilier Guards, both wearing many medals. The following day his Majesty laid the corner stone of a new dock, planted a tree, and reviewed the troops with whom he expressed himself much pleased. On Good Friday evening, while driving to the governor's cottage, his official summer residence, his Majesty stopped to visit the Loreto Convent, where the girls presented him with a bouquet and the King conversed with the nuns, and showed a keen interest in the Asylum of the Little Sisters whose inmates were grouped around. On Monday morning the *Victoria and Albert* left Gibraltar for Malta, and a cruiser and a torpedo boat proceeded, by order, to some point off Algiers, so that wireless telegraphic messages might be exchanged with his Majesty's yacht, which is expected to arrive on Thursday morning. The King is due in Paris about May 1st.

THE Queen is still in Denmark. Last Friday she and her sister, the Dowager-Empress of Russia, paid a visit to the Gule Palace, the residence of Prince and Princess Waldemar. Hearing that the children of the Prince and Princess were at lessons the royal visitors went into the schoolroom, and Queen Alexandra was greatly interested in noting the changes which had been made in the room since she was a pupil. She and her sister were busy recalling the original whereabouts of different pieces of furniture—about which they could not quite agree—when Princess Waldemar intervened and laughingly remarking that she could not have the children's lessons interrupted any longer, carried her guests away. They threatened to bring King Christian with them next time to decide the knotty points under consideration. On Tuesday afternoon the Queen, with the Empress Alexander and the Crown Prince of Denmark, paid a visit to Professor Finsen's hospital and laboratory for the light-cure of lupus.

### The New Field-Marshals.

GENERAL SIR GEORGE WHITE, V.C., and General Sir Evelyn Wood, V.C., have been created field-marshals. The announcement of the advance of the former to the most exalted rank in the Army was made by the King during his visit to Gibraltar. His Majesty, in the course of the State banquet, intimated that he had promoted Sir George White to the dignity of field-marshal, and warmly eulogised his sterling qualities as a soldier, and his long and distinguished record in the past. He said that there had been no more loyal soldier to his Sovereign and country, and that no appointment could be more popular either at Gibraltar or in the Army at large. Sir George White, who is a native of County Antrim, Ireland, and was born in 1835, has served as Commander-in-Chief in India, and is, perhaps, best of all known as the hero of Ladysmith. Sir Evelyn Wood, who was born in 1838 at Cressing, Braintree, Essex, fought with the Naval Brigade at Inkerman, won the Victoria Cross in the Indian Mutiny, distinguished himself in the Ashanti, Kaffir, Zulu, and Transvaal Wars, served in the Nile Expedition, and has been Quastermaster-General and Adjutant-General to the Forces. He has been called to the Bar at the Middle Temple and is an author of some repute. He now commands the Second Army Corps at Salisbury.

### The Late Mrs. Arthur Brand.

THE Hon. Mrs. Arthur Brand, whose remains were laid to rest on Tuesday at the pretty village of Glynde, in Sussex, died under pathetic circumstances at Crawley Downs last week. She had been out driving on Wednesday and just before four o'clock had been watching her only child, a boy of 13, play football with the footman. She then strolled into the wood, and at six o'clock the servants, who had come out to look for her as she had not returned to tea, found her lying dead a few yards from the path in the wood. There were no injuries of any kind, and at the subsequent inquest it transpired beyond doubt that her death was due to a weak heart. Mrs. Brand, who was only 40 years old, was the wife of Mr. Arthur Brand, M.P., who represents the Wisbech division of Cambridgeshire, and to her influence he largely owed his return on four occasions to Parliament. She used to attend his meetings, sing charming songs to the electors and accompanying the songs on the zither. At the last general election her carriage was filled with flowers by the villagers.

### Pictures at the Dudley Gallery.

THE New English Art Club is no longer "new" except in name, for the present exhibition at the Dudley Gallery is the thirtieth which it has held. Most of the exhibits are as daring and as unorthodox as they ever were, but on the whole the collection is a particularly good one, and there is much which, to an artistic temperament, repays a visit. The most important, because the largest, picture is "Mrs. Oliver, Mark and Betty" by Mr. Furse. It is an extremely graceful group, almost life-size, of a mother and children, a somewhat cold arrangement of blue and white. There is a good deal in the treatment of the subject which suggests Mr. Sargent. The Canadian artist, Mr. Homer Watson, sends a couple of pictures, and Mr. Wilson Steer, whose name is inseparably associated with the club, is represented by a water-colour, a decorative design, and an oil landscape, "The Golden Valley," but none are especially remarkable. Mr. Rothenstein's "Doll's House" will probably attract considerable attention, not only because of its ability, but because neither the design nor the allusion to the title is easily understood by the onlooker. "Mother and Child," by Mr. William Orpen, is a really beautiful study in three chalks; "The Red Scarf," by the same painter, is a portrait showing much talent; and "Reflections: China and Japan" is a "still life" picture representing a collection of Chinese and Japanese curios as reflected on the surface of a highly polished table. Pictures of note are also exhibited by Mr. Muirhead, Mr. Horace Livens, Mr. Mark Fisher, and Mr. Hugh Carter.

### A Tram Tour.

IT is now possible to make a complete tour of the picturesque country in the Thames Valley by tramcar. During the Easter holidays the cars made an uninterrupted tour of the loop, and took passengers from Shepherd's Bush or Hammersmith Broadway *viâ* Teddington and Kingston Bridge Approach to Hampton Court Palace, and brought them back again through Hampton, Twickenham and Kew, a distance of 24 miles, without a change of car, and for the low price of 1s. Starts were made every two minutes, and those who secured seats on the outside of the handsome cars had a wonderful diversity of scenery, changing from shop-lined streets in closely-populated suburbs to long roads with fruit trees in full bloom on either side, and except for the inclemency of the weather at times, they must have spent a health-giving holiday. The thousands who crammed the cars from Thursday to Tuesday augurs well for the popularity of the venture during the summer months.

### Notes and Queries.

**The Editor is always willing to answer in this column, without any fee, all reasonable questions, as soon as possible.**

**But the following rules must be carefully observed:—**

1. **Every communication must be accompanied by the name and address of the writer.**
2. **The question must always bear upon nursing, directly or indirectly.**

**If an answer is required by letter a fee of half-a-crown must be enclosed with the note containing the inquiry, and we cannot undertake to forward letters addressed to correspondents making inquiries. It is therefore requested that our readers will not enclose either a stamp or a stamped envelope.**

*Alexandria.*

(15) *G. M. C.*, in reply to Query 185, wishes to state that there is an English hospital, "The Deaconesses," in Alexandria.

*Hospital Service Book.*

(16) Can you kindly tell me of a good service-book for hospital wards?—*Sister F.*

There are two books reviewed in our columns to-day, either of which may serve your purpose.

*Training.*

(17) I thought that perhaps you would kindly give me advice as to training. I am 28 years of age, and thought of training at either the London or Guy's Hospital. I have the particulars of both, but at Guy's I see that the training is for three years, whilst at the London the training is two years with a further two years' service to the hospital. I have read that it is necessary to have a three years' certificate, and do not understand if the certificate from the London would be for two or for four years.—*Perplexed.*

The certificate is given after a training of four years at the London Hospital.

*L.O.S.*

(18) I am a trained maternity nurse, and should like to take the L.O.S. Will you tell me if I must attend classes in theory again, or if it will be sufficient to undergo the practical part of the work only?—*L. W.*

You will certainly have to be taught theory. The Secretary, the Midwives' Institute, 12 Buckingham Street Strand, W.C., would be able to advise you as to your best course.

*Cold Baths.*

(19) Can you tell me where I can get information as to the advantages and disadvantages of cold baths? I am 21, and have had three months' training at a children's hospital. Could I get into the Lincoln County, or any other large general hospital at that age?—*Nurse Hilda.*

You will find the advantages of cold bathing fully discussed in any handbook on hydropathy, but in any case of doubt a medical man should be consulted. The age at which probationers are admitted to the Lincoln County—and most other large general hospitals—is from 23 and upwards.

*Nursing Homes.*

(20) Will you kindly give me the address of private nursing homes in Lincoln?—*Enquirer.*

There is a nursing association managed by a committee, of which the address is Institution for Nurses, Lincoln.

*Massage.*

(21) I am an obstetric nurse, and when opportunity occurs I wish to take a course of lessons in massage, and also in dispensing. Will you kindly tell me how I can best do this?—*Sister Dora.*

You ought to have enclosed your name and address. See our rules to correspondents. You can take a course of massage at the National Hospital for the Paralysed and Epileptic, Queen's Square, Bloomsbury, W.C. Dispensing is, however, difficult, and you must be prepared to devote considerable time to make it of any use. Write to the Secretary, the Pharmaceutical Society, 17 Bloomsbury Square, W.C., for particulars.

*Perspiration.*

(22) Can you recommend any outward application for alleviating unpleasant perspiration of the face, it is very annoying.—*M. R.*

Consult a medical man.

*Auxiliary Nurse Society.*

(23) Will you kindly let me know for what purpose the Auxiliary Nurse Society has been instituted?—*One in Doubt.*

The Auxiliary Nurse Society has been instituted in connection with the Royal British Nurses' Association in order to provide suitable work for the older members of the Association. Particulars may be obtained from the Secretary R.B.N.A., 10 Orchard Street, W.

*Infectious.*

(24) Is a discharging tumour infectious, and is it right to sleep in the same room with the patient? I am nursing a lady under these conditions and should like to know.—*Anxious One.*

This is an important nursing question, and if you have no experience you should take instructions from the doctors.

*Home.*

(25) Will you kindly tell me if there are any homes where a consumptive patient could be received for a moderate charge—say a guinea weekly—and also where I could get information about them.—*Nurse E. D.*

There are several homes of which you may obtain particulars by consulting "Burdett's Hospitals and Charities."

*Maternity Case.*

(26) I was engaged in January for August. In consequence refused two cases. The case miscarried, and I was not sent for. Can I claim compensation? And if I had been sent for and not been free to go could I *legally* claim?—*C.E.S.*

Legally you cannot make a claim. You should always arrange by letter for these contingencies when making an engagement. It is usual to receive half the fee in such cases.

*Books.*

(27) Will you kindly tell me if there is any other paper besides The Hospital which advertises for nurses seeking appointments? And, if so, where can I get it?—*A. B.*

Advertisements for and by nurses appear in the medical journals, and in the daily papers, but The Hospital is the best medium, as you will find by experience.

*Matron's Duties, Housekeeping, etc.*

(28) Will you please tell me the name of any book on "Matron's Duties, Housekeeping," etc.?

"Hospital Expenditure: the Commissariat," published by the Scientific Press (2s. 6d.), is a most useful volume for any matron, but there is no comprehensive work on the subject at present.

*Algiers.*

(29) Will you kindly let me know if there is any address in London to which I could apply for a nursing post in Algiers?—*Nurse G.*

There is no association for supplying nurses in Algiers. You might hear of private work through the matron of your training school. Algiers being a French colony, there is not the same facilities for the employment of English nurses as in our own Crown Colonies.

*M. A. V.*

(30) Will you kindly tell me who the M. A. V. nurses are, and what are the rules. Also can you name a reliable nurses' co-operative nurses' association besides that of 8 New Cavendish Street.—*I. N.*

We have not heard of the M. A. V. nurses. The Society of Chartered Nurses, 24 Princes Street, Cavendish Square, W., adopt the same principles.

*Infirmary Training.*

(31) Will you kindly tell me if the training at the large provincial workhouse infirmaries—such as that at Birmingham—is accounted good, and if it would qualify for admission to the Navy Nursing Staff?—*Constance.*

The training at the larger infirmaries, Birmingham being one of the most notable, is highly esteemed. If, however, you are desirous of entering the Navy Nursing Staff, we should recommend you to write to the Director-General, Medical Department of the Navy, Northumberland Avenue, W.C., and ask if the school at which you propose training, fulfils the conditions.

*British Gynæcological Examination for Nurses.*

(32) Can you tell me a useful book for a nurse to study who wishes to go in for the above examination?—*A. L.*

"Gynæcological Nursing," by G. A. Hawkins-Ambler, published by the Scientific Press (1s.), is a useful elementary work.

**Important Nursing Textbooks.**

"The Nursing Profession: How and where to Train." 2s. net; 2s. 4d. post free.

"A Handbook for Nurses." (New Edition). 5s. net; 5s. 4d. post free.

"The Human Body." 5s. post free.

"Ophthalmic Nursing." (New Edition). 3s. 6d. net; 3s. 10d. post free.

"Gynæcological Nursing." 1s. post free.

"Art of Feeding the Invalid." (Popular Edition). 1s. 6d. post free.

"Practical Hints on District Nursing." 1s. post free.

